# Modified Sanjia Powder ameliorates cognitive impairment and exerts neuroprotective effects in 5 × FAD mice: insights from quantitative proteomics

**DOI:** 10.3389/fnut.2025.1699142

**Published:** 2026-01-16

**Authors:** Yifan Wang, Chenxi Liu, Xiaoyan Zheng, Zhiqiang Wang, Yufan Wang, Yi Geng, Jin Yang, Kaifeng Wei, Xiaoying Chen

**Affiliations:** 1 School of Chinese Medicine, Nanjing University of Chinese Medicine, Nanjing, China; 2 Academy of First Clinical Medicine, Nanjing University of Chinese Medicine, Nanjing, China; 3 School of Integrated Chinese and Western Medicine, Nanjing University of Chinese Medicine, Nanjing, China; 4 School of Medicine, Nanjing University of Chinese Medicine, Nanjing, China; 5 Affiliated Hospital of Nanjing University of Chinese Medicine, Jiangsu Province Hospital of Chinese Medicine, Nanjing, China

**Keywords:** 5 × FAD mice, Alzheimer's disease, cognition, Modified Sanjia Powder, neuroprotective

## Abstract

**Background:**

Modified Sanjia Powder (MSP) is a traditional Chinese herbal formulation with potential use as a dietary supplement, which has shown neuroprotective properties against Alzheimer's disease (AD). However, its mechanisms of action, particularly those related to metabolic pathways, remain poorly understood. Given the emerging role of lipid metabolism and associated oxidative stress in AD pathogenesis, this study aimed to investigate the therapeutic effects of MSP on cognitive impairment and explore its molecular mechanisms, with emphasis on nutritionally relevant pathways, in the 5 × FAD mouse model of AD using quantitative proteomics.

**Methods:**

Cognitive, pathological, and molecular functions were evaluated following MSP treatment. Cognitive performance was assessed using behavioral tests including the Y-maze, novel object recognition (NOR), and Morris Water Maze. Brain tissues from control, 5 × FAD, and MSP-treated mice were analyzed by data-independent acquisition mass spectrometry to identify differentially expressed proteins (DEPs). Key findings were validated using Western blotting, immunohistochemistry, and cytokine assays.

**Results:**

MSP treatment significantly improved cognitive function in 5 × FAD mice across multiple behavioral tests. It reduced Aβ plaque deposition, attenuated tau hyperphosphorylation, inhibited microglial activation, and decreased levels of pro-inflammatory cytokines (IL-1β, TNF-α, and IL-6). Proteomic analysis identified 460 DEPs, with significant enrichment in pathways related to fatty acid biosynthesis, lipid metabolism, and oxidative stress. Notably, among these DEPs, ACSL4—a key regulator of lipid metabolism and oxidative stress—was upregulated in 5 × FAD mice but markedly downregulated after MSP treatment. Importantly, MSP's modulation of lipid metabolism appeared selective for the ACSL4 pathway, without broadly affecting other lipid metabolic pathways that influence cytokine release. MSP also reduced levels of reactive oxygen species (ROS) and lipid peroxidation markers (MDA and 4-HNE).

**Conclusion:**

MSP confers neuroprotection in AD by modulating ACSL4-mediated lipid metabolism and oxidative stress, leading to improved cognitive function and reduced neuroinflammation in the 5 × FAD mouse model. These results position MSP as a promising therapeutic candidate for AD and demonstrate the value of quantitative proteomics in elucidating the mechanisms of traditional Chinese medicines.

## 1 Introduction

Alzheimer's disease (AD) is a progressive neurodegenerative disorder that predominantly affects the elderly population. Currently, an estimated 50 million people live with dementia globally, a number projected to triple by 2050, positioning AD among the most pressing public health issues of this century (1). Clinically, AD manifests as cognitive decline, memory loss, and progressive impairment in daily activities, underpinned by the accumulation of amyloid-beta (Aβ) plaques and neurofibrillary tau tangles. These pathological hallmarks disrupt synaptic plasticity, neuronal communication, and blood–brain barrier integrity, ultimately leading to synaptic failure and neuronal death (2, 3). Despite decades of research, no disease-modifying therapies are available; current treatments provide only symptomatic relief (4), underscoring the critical need for novel interventions that target fundamental disease mechanisms.

Growing evidence emphasizes the central role of disrupted lipid metabolism in AD pathogenesis. Indeed, lipidomic studies have revealed substantial remodeling of brain phospholipid acyl-chains associated with APOEε4, the strongest genetic risk factor for AD, and mild cognitive impairment (5–7). Among the enzymes governing this lipid landscape, the acyl-CoA synthetase (ACSL) family plays a critical role in determining brain phospholipid acyl-chain diversity by channeling fatty acids into various metabolic pathways (8). Specifically, acyl-CoA synthetase long-chain family member 4 (ACSL4) has emerged as a key regulator linking lipid dyshomeostasis to AD pathology (9, 10). Recent studies have shown that ACSL4-driven accumulation of lipid droplets in microglia is closely associated with APOE4, the most potent genetic risk factor for AD. APOE4 promotes the influx of fatty acids into glial cells, which, through ACSL1 (a homolog of ACSL4)-mediated pathways, leads to lipid droplet formation and neurotoxicity (11). ACSL4 catalyzes the biosynthesis of long-chain fatty acyl-CoA derivatives, critical for maintaining neuronal membrane integrity and synaptic function in the brain (12, 13). In AD, ACSL4 dysfunction triggers a self-reinforcing pathological cascade through dysregulated polyunsaturated fatty acid (PUFA) metabolism, leading to excessive lipid peroxidation and subsequent accumulation of 4-hydroxynonenal (4-HNE), a reactive aldehyde that covalently cross-links Aβ peptides and stabilizes hyperphosphorylated tau aggregates (14, 15). Concurrently, these cytotoxic metabolites activate the NF-κB and NLRP3 inflammasome pathways, creating a feed-forward loop that perpetuates neuroinflammation while suppressing key Aβ-degrading enzymes (16, 17). Notably, ACSL4 also heightens neuronal susceptibility to ferroptosis, an iron-dependent form of cell death linked to antioxidant depletion and oxidative membrane damage (10, 18). Thus, among the redundant ACSL and acyltransferase enzymes, ACSL4 is prioritized due to its unique substrate preference for pro-inflammatory, positioning it as a promising therapeutic target to slow AD progression.

Within the framework of “medicine-food homology”, Traditional Chinese Medicine (TCM) offers a rich source of dietary supplements with potential neuroprotective benefits. Modified Sanjia Powder (MSP) is one such formula, which has demonstrated anti-inflammatory and neuroprotective properties in preclinical AD models (19, 20). Previous studies indicate that MSP reduces Aβ accumulation and suppresses the secretion of pro-inflammatory cytokines such as IL-1α, IL-1β, and IL-6, thereby ameliorating neuronal damage in the hippocampus (21–23). However, despite these promising effects, it remains unclear whether MSP modulates ACSL4-related lipid metabolic pathways and oxidative stress responses. Moreover, whether its effects are selective for the ACSL4 pathway amidst the broader landscape of lipid metabolism remains unknown. This knowledge gap limits its development as a standardized dietary intervention for AD.

Quantitative proteomics has emerged as a powerful tool for elucidating complex mechanisms underlying natural products (24, 25), and has been increasingly applied to investigate TCM formulas (26, 27). In this study, we employed quantitative proteomics to explore the effects of MSP on the brain proteome of 5 × FAD mice, a well-established model of AD, with a focus on ACSL4-mediated lipid metabolism and oxidative stress. Our unbiased proteomic screen identified ACSL4 as a key node within the significantly enriched lipid and oxidative stress pathways, providing a data-driven rationale for its prioritization for further mechanistic investigation. Our goal is to decipher the molecular mechanisms through which MSP exerts its protective effects, thereby bridging traditional medicine with contemporary nutritional neuroscience and facilitating the development of evidence-based, diet-compatible therapies for AD.

## 2 Materials and methods

### 2.1 Preparation and standardization of MSP

MSP is a traditional formulation composed of the following six components, all sourced from authenticated biological and herbal origins: Trionycis Carapax (20 g; carapace of Trionyx sinensis Wiegmann), Eupolyphaga Steleophaga (10 g; dried female specimens of Eupolyphaga sinensis Walker or Steleophaga plancyi), Testudinis Carapax et Plastrum (20 g; shell of Chinemys reevesii Gray), Pheretima (10 g; dried earthworms including Pheretima aspergillum, *P. vulgaris, P. guillelmi*, or *P. pectinifera*), Polygoni Multiflori Radix (20 g; root of Polygonum multiflorum Thunb.), and Acori Tatarinowii Rhizoma (20 g; rhizome of Acorus tatarinowii Schott). All materials were obtained from Jiangyin Tianjiang Pharmaceutical Co., Ltd. (Jiangyin, China) and were identified by Dr. Chuangfeng Tang, School of Pharmacy, Nanjing University of Chinese Medicine (Nanjing, China). Morphological, microscopic authentications and thin layer chromatography were performed in accordance with Chinese Pharmacopeia (2020). Herbarium voucher specimens of the tested botanical and zoological drugs were deposited at the School of Pharmacy, Nanjing University of Chinese Medicine. All the raw materials were soaked in purified water (1:10, w/v) for 30 min, extracted twice with boiling water for 1 h, and then the supernatant was filtered through a mesh filter with a pore size of 200 μm. The water extracts of MSP were concentrated, freeze-dried and stored at −80 °C. The yield of MSP was about 3.22% (100 g/3.22 g).

The metabolite contents of MSP were detected by Liquid Chromatography-Tandem Mass Spectrometry (LC-MS/MS). The sample was dissolved in 20 mL of ultrapure water. A 200 μL aliquot of the solution was mixed with 800 μL methanol (4:1, v/v), vortexed for 5 min, and centrifuged at 18,000 × *g* for 10 min. The supernatant was filtered through a 0.22 μm nylon membrane prior to injection. Separation was performed on a Waters BEH C18 column (2.1 × 100 mm, 1.7 μm) maintained at 45 °C. The mobile phase consisted of: (A) 0.1% formic acid in water and (B) 0.1% formic acid in acetonitrile, with a flow rate of 0.3 mL/min and an injection volume of 2 μL. Gradient elution was programmed as follows: 0–1 min, 10% B; 1–10 min, 10% → 90% B; 10–12 min, 90% → 10% B; 12–15 min, 10% B. The mass spectrometer was operated in both positive and negative ion modes with the following parameters: spray voltage, +4.0 kV (positive)/−3.5 kV (negative); vaporizer temperature, 300 °C; capillary temperature, 350 °C; sheath gas flow, 45 arb; auxiliary gas flow, 25 arb. Multiple reaction monitoring (MRM) transitions were optimized as detailed in Supplementary Table S1. Mixed reference standards were accurately weighed and dissolved in methanol to prepare stock solutions at appropriate concentrations. Calibration curves were constructed by serial dilution (1:2) of the stock solutions. The metabolite contents of MSP are shown in Supplementary Table S2 and Supplementary Figure S1.

### 2.2 Animal experiments

All animal experiments comply with the ARRIVE guidelines and were carried out in accordance with the U.K. Animals (Scientific Procedures) Act, 1986. All animals were maintained under standard conditions at room temperature (22 °C) under a 12 h light/dark cycle. The animal experiments were approved by the Experimental Animal Ethics Committee of Nanjing University of Chinese Medicine (No. 202008A002). Female wild-type (WT) mice (C57BL/6J background) and 5 × FAD transgenic mice (APPSwFlLon, PSEN1 × M146L × L286V) mice were obtained from a breeding pair purchased from the Jackson Laboratory agency by Nanjing Institute of Biomedicine (Nanjing, China). The 5 × FAD mice carry five familial Alzheimer's disease mutations: APP K670N/M671L (Swedish), I716V (Florida), V717I (London), PS1 M146L, and L286V (28), maintained through the Nanjing Institute of Biomedicine's Specific Pathogen-Free (SPF) facility under strict genetic background control (backcrossed to C57BL/6J for >10 generations). A stratified randomization procedure based on body weight was implemented by a researcher not involved in subsequent behavioral testing and data analysis to assign mice into four groups: Control, 5 × FAD, MSP-L (low dose), and MSP-H (high dose), with eight animals per group. Starting at 7 months of age, 5 × FAD mice in the MSP-L and MSP-H groups were administered MSP via daily intragastric gavage at doses of 15.02 g/kg (crude drug, equivalent to 0.48 g/kg extract) and 30.04 g/kg (crude drug, equivalent to 0.96 g/kg extract), respectively, for 30 consecutive days. The extraction yield of MSP was 3.2%, and the dose conversion between crude drug and extract was calculated using the following equation: Equivalent extract dose (g/kg) = Crude drug dose (g/kg) × Extraction yield (%) (29, 30). All behavioral assessments, histological evaluations, and data analysis were performed by investigators blinded to the group allocation of the animals. At the conclusion of the experiment, mice were euthanized via cervical dislocation, and brain tissue samples were collected and stored for subsequent biochemical and histological analysis.

### 2.3 Behavioral tests and analysis

All behavioral tests were conducted in a sound-attenuated room with controlled lighting (50 lux) and recorded using the Visutrack software (version 3.0, Xinruan Information Technology Co., Ltd.). Mice were habituated to the testing room for at least 30 min prior to each behavioral session.

#### 2.3.1 Y-maze test

The Y-maze apparatus consisted of three V-shaped arms (40 **×** 3 **×** 12 cm), arranged at 120° angles. To assess spontaneous alternation behavior, individual mice were placed at the distal end of one arm and allowed to explore the maze freely for 8 min under low-light conditions (50 lux). Spontaneous alternation was defined as successive entries into all three arms. The sequence of arm visits and total number of entries were recorded, and the alternation ratio was calculated (31, 32).

#### 2.3.2 Novel object recognition (NOR) test

The test followed an established protocol described in previous studies (33). Briefly, the procedure included four consecutive daily trials. During the adaptation phase (Days 1 and 2), mice were positioned in the center of the apparatus and allowed to explore freely for 15 min without the presence of any objects, ensuring familiarity with the environment. In the acquisition phase (Day 3), two identical objects were introduced into the apparatus, and the mice were permitted to explore the space and interact with the objects for 10 min. On Day 4, during the memory test phase, one of the familiar objects was replaced by a novel object with distinct features such as shape, color, and texture. The mice were then allowed to explore both objects freely for 10 min. All data on exploratory behavior were collected for subsequent performance analysis. The discrimination index (DI) was calculated as the difference in exploration time for the novel object (TN) and the familiar object (TF), divided by the total exploration time (TN + TF). As shown in Equation 1:

$$\begin{array}{l} \text{DI}=\;\left[\text{TN}-\text{TF}\right]/\left[\text{TN}+\text{TF}\right] \end{array}$$ (1)

#### 2.3.3 Morris water maze (MWM) test

The MWM test was performed as described previously (34). It included a positioning navigation phase and a spatial probe phase. During the navigation (training) phase, mice were trained to locate a hidden platform, with the MWM system automatically recording their swimming paths and escape latencies. Following five consecutive days of training, escape latency was reassessed. During the probe phase, the platform was removed, and mice were allowed 60 s to search for its previous location. Performance data were recorded for subsequent analysis.

### 2.4 Immunofluorescent staining

Brain tissue sections were treated with phosphate-buffered saline with Tween 20 (PBST) for 15 min, followed by blocking with 5% BSA for 1 h at room temperature. Sections were then incubated overnight at 4 °C with primary antibodies targeting 6E10 (BioLegend, 803004, 1:300) and Iba-1 (Wak, 019-19741; 1:300). After rinsing with PBS, Alexa Fluor 488- and 594-conjugated secondary antibodies (Invitrogen, A-11008 and A-21203, 1:500) were applied for 2 h, and nuclei were counterstained with Hoechst 33342. Slides were examined under a Leica DMI8 fluorescence microscope (Wetzlar, Germany), and images were analyzed using ImageJ software.

### 2.5 AT8 immunohistochemical staining

Brain sections (20 μm) were permeabilized with 5% Triton X-100 for 10 min at room temperature. After blocking in 5% bovine serum albumin (BSA) for 30 min, sections were incubated overnight at 4 °C with the AT8 primary antibody (Thermo Fisher Scientific, MN1020, 1:250). Following three washes with phosphate-buffered saline (PBS), sections were incubated with horseradish peroxidase (HRP)-conjugated secondary antibodies (Abcam, ab6721, 1:500) for 2 h at room temperature. The antibody binding was visualized using a 33′-diaminobenzidine (DAB) solution, producing a brown precipitate. Stained sections were examined and imaged using a Leica DM1000 microscope (Wetzlar, Germany), and images were analyzed for both quantitative and qualitative parameters using ImageJ software.

### 2.6 Enzyme-linked immunosorbent assay (ELISA)

Levels of IL-1β, TNF-α, and IL-6 in brain tissue were measured using commercial ELISA kits (IL-1β: Jiancheng, H002; TNF-α: Jiancheng, H052; IL-6: Jiancheng, H007) according to the manufacturers' instructions. Brain tissues were homogenized in radioimmunoprecipitation assay (RIPA) buffer containing a protease inhibitor cocktail and kept on ice for 30 min. After homogenization, samples were centrifuged at 20,000 × *g* for 30 min at 4 °C. The supernatants were collected, and protein concentration was determined using a Bicinchoninic acid (BCA) protein assay kit (Beyotime Biotech, Shanghai, China).

### 2.7 Proteomic analysis and data acquisition

For proteomic analysis, brain tissue samples from 3 randomly selected mice per group (*n* = 3) were processed. Brain tissue samples were homogenized in RIPA buffer (Sigma-Aldrich) supplemented with a protease inhibitor cocktail (Roche) on ice for 30 min. Protein concentrations were determined using the BCA Protein Assay Kit (Beyotime Biotech). The proteins were reduced with dithiothreitol (DTT) and alkylated with iodoacetamide (IAA). Proteins were then digested overnight at 37 °C with trypsin (Promega, V5111) at a 1:50 enzyme-to-protein ratio. Peptides were desalted using a C18 solid phase extraction (SPE) column (Waters) and lyophilized before liquid chromatography-tandem mass spectrometry (LC-MS/MS) analysis. LC-MS/MS was performed using a NanoElute liquid chromatography system (Bruker Daltonics Inc.) coupled with a hybrid Trapped Ion Mobility Spectrometry (TIMS) quadrupole time-of-flight (TOF) mass spectrometer (Bruker TimsTOF Pro). Peptides were separated on a 75 μm **×** 25 cm ReproSil-Pur C18 column (Dr. Maisch HPLC GmbH) with a 120-min gradient of 5–35% acetonitrile in 0.1% formic acid at a flow rate of 300 nL/min. Data analysis was performed using DIA-NN software (version 1.8.1, https://github.com/vdemichev/DiaNN), with a false discovery rate (FDR) < 1% and a minimum of two peptide identifications per protein. The analysis was conducted in library-free mode, with default deep learning-based spectral and retention time prediction enabled. The search parameters included trypsin digestion specificity with a maximum of two missed cleavages, fixed modification of carbamidomethylation on cysteine residues, and variable modification of methionine oxidation. Default settings were employed unless otherwise specified. Precursor and fragment ion mass accuracies were both set to 20 ppm. The “robust” protein inference strategy was used, and match-between-runs (MBR) was enabled to enhance quantification consistency across samples. Protein and metabolite identification were based on the Mus musculus UniProt database (UniProt accession: Mus musculus-10090-2019-10), containing reviewed canonical sequences. Proteins with >60% quantitative information were included in further analysis, and missing values were imputed using the k-nearest neighbors (KNN) algorithm, implemented via the impute package in R. Data were normalized using the median method and log2-transformed to account for loading differences. Statistical analysis was performed using R (version 4.0.2), and a single-factor ANOVA followed by Tukey's test for pairwise comparisons was applied. Fold Change (FC) was calculated as the ratio of protein abundance between groups, with FC >1.1 or < 0.91 and *p* < 0.05 as the thresholds for significant changes. Bioinformatics analyses, including Gene Ontology (GO) and Kyoto Encyclopedia of Genes and Genomes (KEGG) pathway enrichment, were performed using Metascape (https://metascape.org) with a *p*-value threshold of < 0.05 and FDR correction for multiple testing.

### 2.8 Western blot analyses

For brain samples, tissues were homogenized with RIPA buffer supplemented with protease and phosphatase inhibitor cocktails, and the homogenates were kept on ice for 30 min. Supernatants were collected by centrifugation at 12,000 **×***g* for 15 min at 4 °C. Protein samples were mixed with loading buffer (Thermo Scientific) and boiled for 15 min at 95 °C. Proteins were separated by SDS-PAGE and transferred to a nitrocellulose membrane (GE Healthcare) using a Mini-PROTEAN Tetra Gel Electrophoresis System (Bio-Rad, CA, USA). After blocking for 2 h at room temperature, the membranes were incubated overnight at 4 °C with primary antibodies against ACSL4 (Proteintech, 22401-1-AP, 1:2000) and β-actin (Proteintech, 60008-1-Ig, 1:5000). After washing thrice, the membranes were incubated with HRP-conjugated secondary antibodies (Proteintech, SA00001-1 and SA00001-2, 1:5000) for 2 h at room temperature. Blots were developed and visualized using a Gel Imaging System (Tanon 5200, Tanon, Shanghai, China).

### 2.9 Measurement of reactive oxygen species (ROS) production

Mouse brain tissues were homogenized in isotonic buffer (10 mM Hepes, 200 mM mannitol, 70 mM sucrose, 1 mM EDTA pH 7.6, 1% NP40, 1X protease inhibitor cocktail) at a 1:10 tissue weight/lysis buffer volume ratio using a Potter homogenizer with 30 strokes at 1000 rpm, kept on ice. The total protein concentration was determined using the Bio- Rad DC protein assay (Bio-Rad Laboratories, Hercules, CA, USA). ROS production was measured using the ROS-ID^®^ Total ROS/Superoxide Detection Kit (Enzo Life Sciences, catalog no. ENZ-51010) according to the manufacturer's instructions. Fluorescence was detected with a TECAN Infinite F200 Pro Plate Reader (Tecan Group, Männerdorf, Switzerland) using fluorescein (excitation at 485 nm, emission at 535 nm) and rhodamine filters (excitation at 540 nm, emission at 590 nm). Data are presented as a percentage (%) of control (35).

### 2.10 Measurement of malondialdehyde (MDA) and 4-hydroxynonenal (4-HNE) levels

MDA and 4-HNE levels were assessed using the Lipid Peroxidation (MDA) Assay Kit (Sigma-Aldrich, MAK085) and Lipid Peroxidation (4-HNE) Assay Kit (Abcam, ab238538), following the manufacturer's instructions.

### 2.11 Statistical analysis

Statistical analyses were performed using GraphPad Prism 8.0 software (San Diego, CA, USA). All data are presented as the mean ± SEM and were tested for normality and homogeneity of variance. Comparisons between two groups were performed using an unpaired *t*-test, while comparisons among multiple groups were analyzed using one-way ANOVA. A *p*-value < 0.05 was considered statistically significant.

## 3 Results

### 3.1 MSP attenuates cognitive impairments in 5 × FAD mice

**Figure 1:**
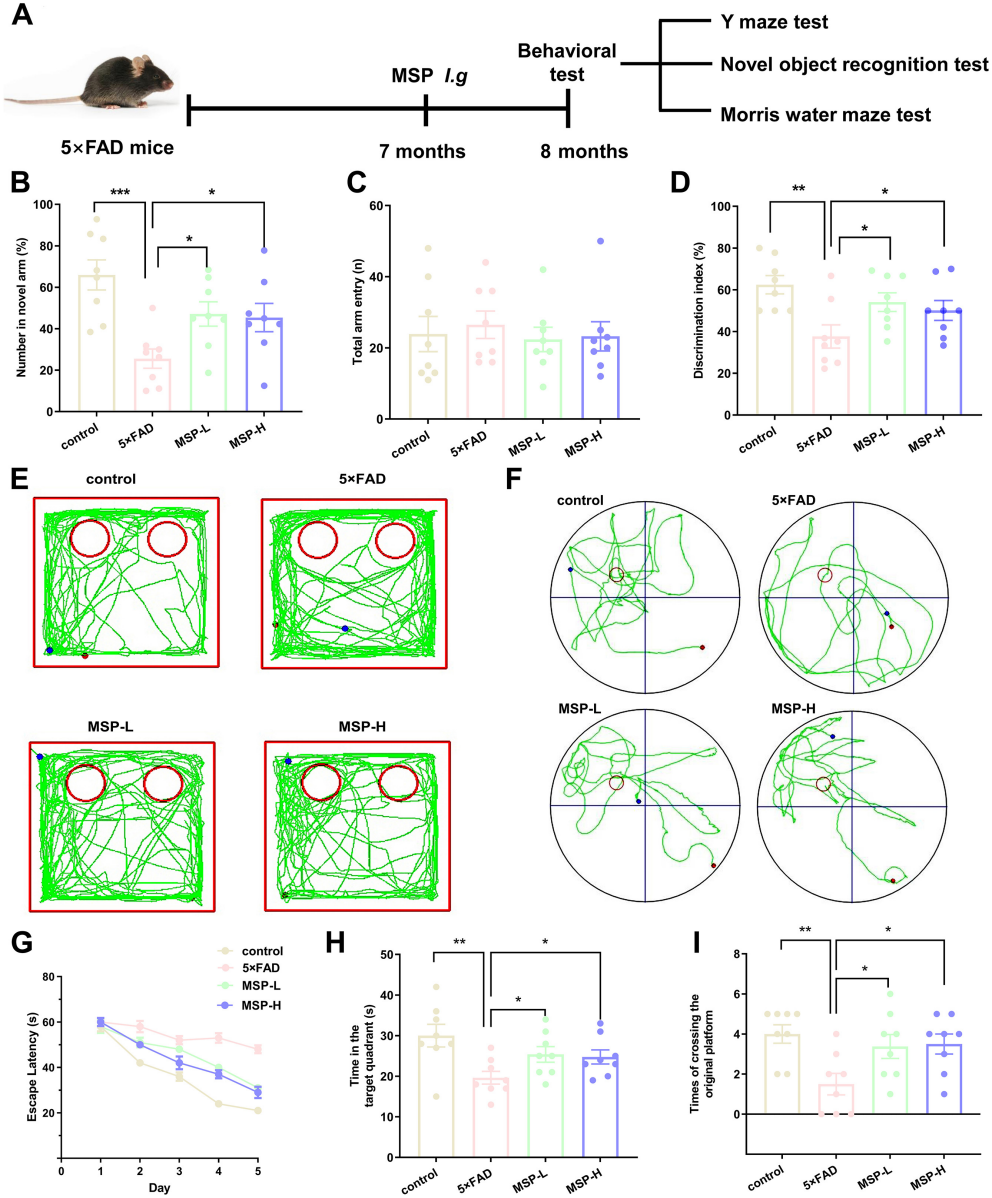
MSP treatment mitigates cognitive deficits in 5 × FAD mice. **(A)** Experimental timeline for the 5**×**FAD mouse model, including treatment and behavioral testing. **(B)** Cognitive flexibility was assessed using the Y-maze test, with the percentage of alternations recorded. **(C)** Total arm entries in the Y-maze. **(D)** Representative tracing graphs in novel object recognition (NOR) test. **(E)** Quantitative analysis of the NOR test, indicating recognition memory performance. **(F)** Representative tracing graphs in Morris Water Maze (MWM) test. **(G)** Escape latency during the MWM training, measuring time to find the hidden platform. **(H)** Number of entries into the target quadrant during the probe trial, reflecting spatial memory. **(I)** Number of platform crossings during the probe trial, indicating memory retrieval. MSP-L and MSP-H denote low-dose and high-dose MSP treatment, respectively. Data are presented as mean ± SEM (*n* = 8 per group). *p < 0.05, **p < 0.01, ***p < 0.001.

Cognitive function was assessed in 5 × FAD mice using the Y-maze, novel object recognition (NOR), and Morris Water Maze (MWM) tests, which are commonly used to evaluate cognitive deficits in neurodegenerative disease models (36). The experimental design is outlined in Figure 1A. In the Y-maze novel arm test, 5 × FAD mice made significantly fewer entries into the novel arm compared to control mice, indicating impaired spatial working memory. After treatment with either low-dose (MSP-L, 15.02 g/kg crude drug) or high-dose (MSP-H, 30.04 g/kg crude drug) MSP, the number of novel arm explorations increased significantly, suggesting that MSP improved spatial working memory in 5 × FAD mice (Figures 1B, C). In the NOR test, 5 × FAD mice demonstrated a preference for sniffing the familiar object, indicating novelty recognition deficits. Both doses of MSP treatment significantly increased exploration time of the novel object, reflecting improved short-term memory (Figures 1D, E). During the MWM 5-day training phase, 5 × FAD mice exhibited prolonged escape latency compared to controls. Both MSP-L and MSP-H MSP treatment significantly reduced latency. In the probe test, 5 × FAD mice spent less time in the target quadrant and made fewer platform crossings. These deficits were reversed by MSP (Figures 1F–I), demonstrating improved spatial learning and long-term memory. Notably, no significant differences were observed between the MSP-L and MSP-H groups across all behavioral tests, indicating comparable efficacy at both doses. Overall, these findings suggest that MSP effectively mitigates cognitive impairments in the 5 × FAD mouse model of AD.

### 3.2 MSP reduces Aβ plaques and tau pathology

**Figure 2:**
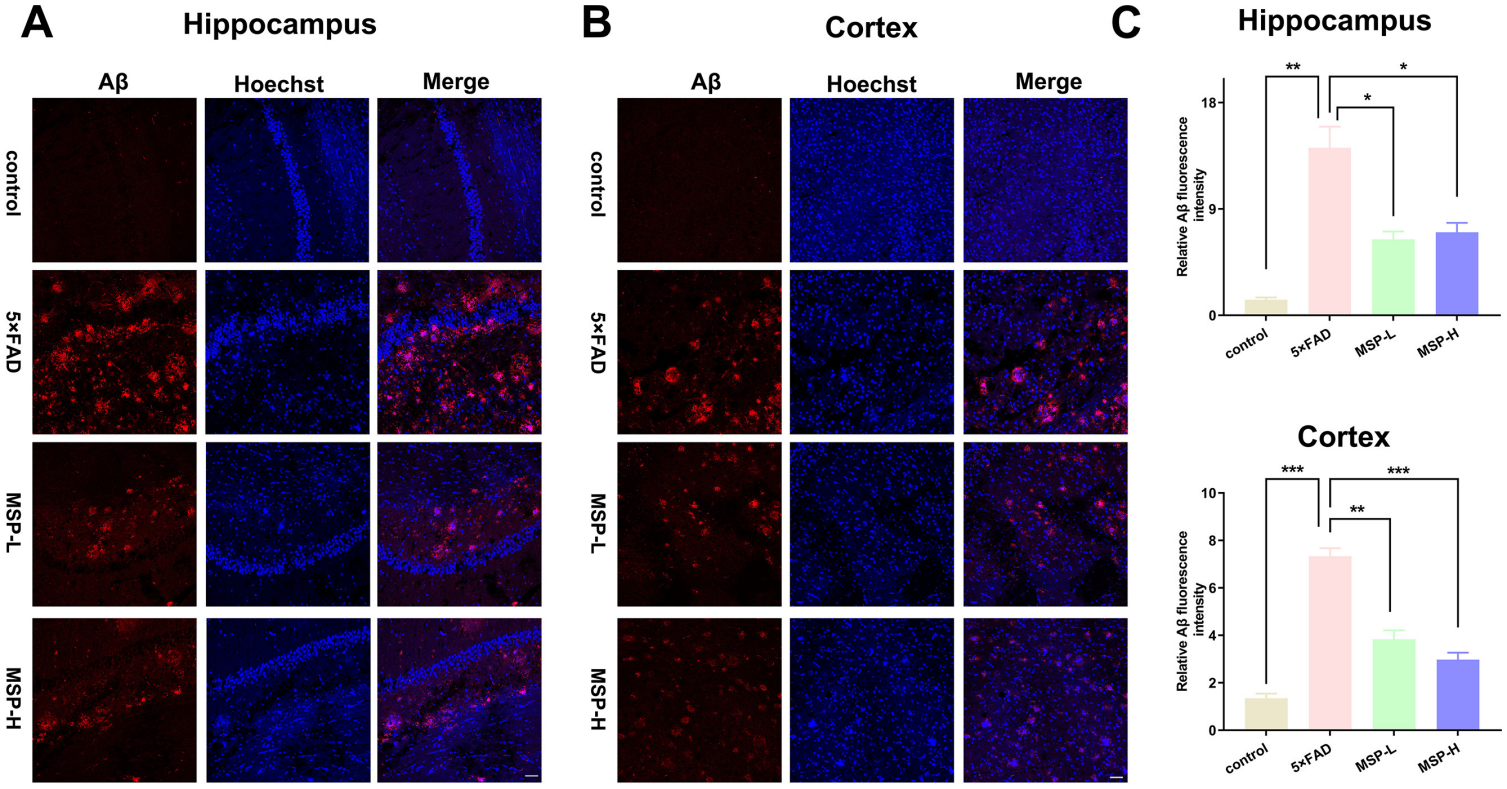
MSP treatment reduces Aβ plaques in 5 × FAD mice. **(A, B)** Immunofluorescence images showing Aβ plaques in the brain of 5 × FAD mice. **(C)** Quantitative analysis of Aβ plaque accumulation in the brain. Scale bar = 100 μm. MSP-L and MSP-H denote low-dose and high-dose MSP treatment, respectively. Data are presented as mean ± SEM (*n* = 8 per group). *p < 0.05, **p < 0.01, ***p < 0.001.

**Figure 3:**
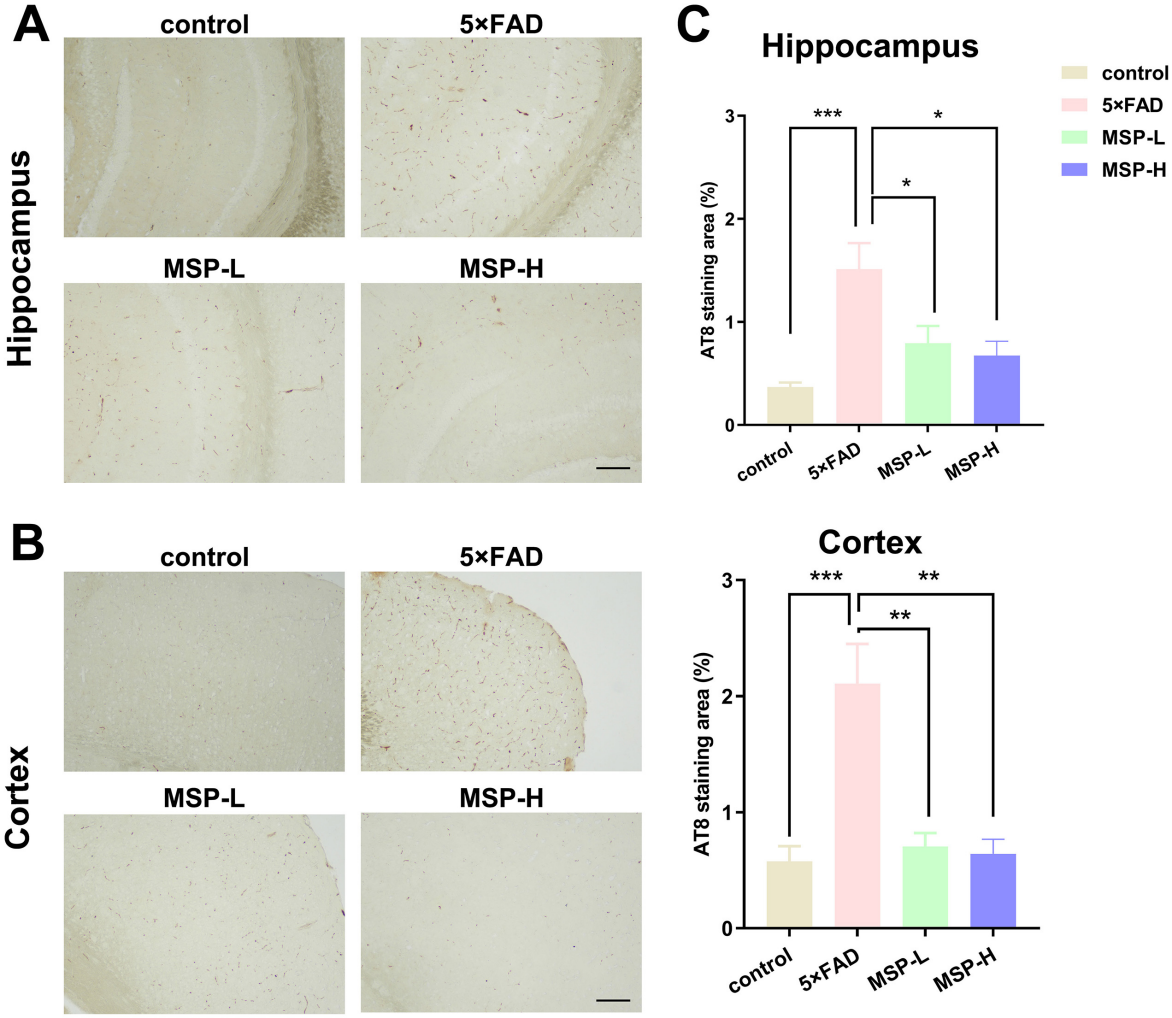
MSP treatment reduces tau hyperphosphorylation in 5 × FAD mice. **(A, B)** AT8 staining showing tau hyperphosphorylation in the brains of 5 × FAD mice. **(C)** Quantitative analysis demonstrating that MSP treatment significantly reduces tau hyperphosphorylation in the brain. Scale bar = 100 μm. MSP-L and MSP-H denote low-dose and high-dose MSP treatment, respectively. Data are presented as mean ± SEM (*n* = 8 per group). *p < 0.05, **p < 0.01, ***p < 0.001.

Aβ plaques and tau hyperphosphorylation are key pathological hallmarks of AD (37). To assess the effects of MSP on these hallmarks, we first analyzed Aβ deposition and tau pathology. Immunofluorescence staining revealed a dense distribution of Aβ plaques in the hippocampus and cortex of 5 × FAD mice, in contrast to control mice. Following low-dose and high-dose MSP treatment, the number of Aβ plaques was significantly reduced (Figure 2). Furthermore, DAB staining for tau hyperphosphorylation using the AT8 antibody showed a significant increase in AT8-positive areas in the hippocampus and cortex of 5 × FAD mice compared to control mice. Following low-dose and high-dose MSP treatment, AT8-positive staining was significantly reduced (Figure 3). These results suggest that MSP alleviates both Aβ deposition and tau pathology in 5 × FAD mice.

### 3.3 MSP inhibits microglial overactivation and neuroinflammation

**Figure 4:**
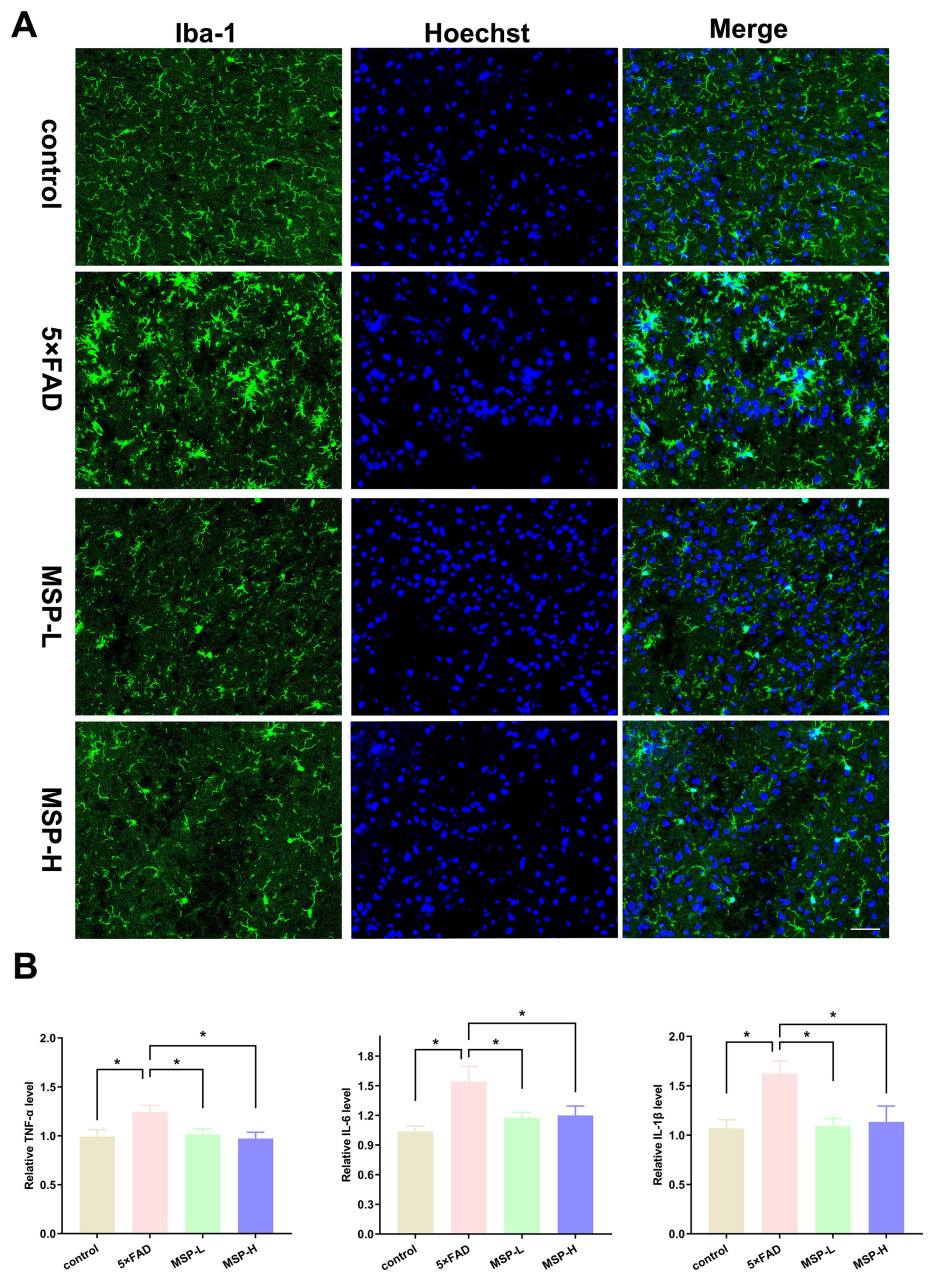
MSP treatment inhibits microglial overactivation and neuroinflammation in 5 × FAD mice. **(A)** Immunofluorescence images showing Iba-1 expression in the hippocampus of 5 × FAD mice. **(B)** Proinflammatory cytokine levels (TNF-α, IL-1β, and IL-6) in the hippocampus of 5 × FAD mice. Scale bar = 100 μm. MSP-L and MSP-H denote low-dose and high-dose MSP treatment, respectively. Data ware presented as mean ± SEM (*n* = 8 per group). *p < 0.05.

Excessive microglia activation contributes to neuroinflammation in AD by impairing Aβ clearance, inducing neurotoxicity, and accelerating disease progression (38, 39). To determine whether MSP modulates microglial activation and neuroinflammation, we examined the activation marker Iba-1 and levels of pro-inflammatory cytokine. Immunofluorescence staining revealed a significant increase in Iba-1 fluorescence intensity in the hippocampus of 5 × FAD mice, indicating microglial activation. Both low-dose and high-dose MSP treatment effectively reduced Iba-1 intensity (Figure 4A), suggesting that MSP attenuates microglial activation. Furthermore, analysis of inflammatory cytokines showed that both low-dose and high-dose MSP significantly reduced the levels of pro-inflammatory cytokines, including TNF-α, IL-6, and IL-1β, in the hippocampus of 5 × FAD mice (Figure 4B). These findings demonstrate that MSP inhibits microglial overactivation and reduces neuroinflammation in the 5 × FAD mouse model of AD.

### 3.4 Enrichment analysis of differentially expressed proteins (DEPs) in response to MSP

**Figure 5:**
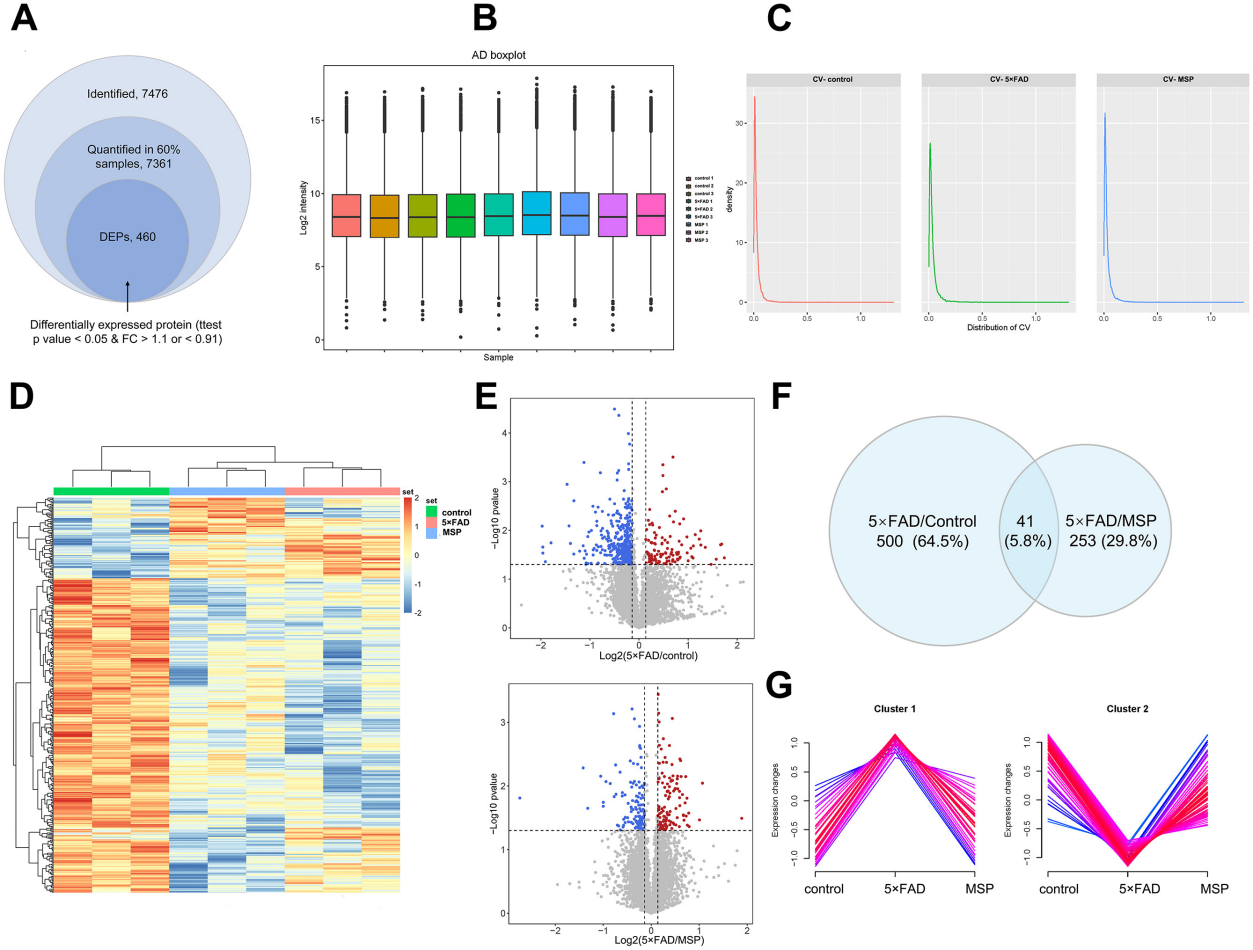
Proteomic analysis hippocampal tissues from control, 5 × FAD, and MSP-treated mice. **(A)** Summary of identified and quantified proteins, with 460 differentially expressed proteins (DEPs) found between the MSP-treated and control groups when compared to the 5**×**FAD group. **(B)** Boxplot illustrating the protein and metabolite profiles across the three groups. **(C)** Density distribution of coefficient of variation in the three groups. **(D)** Clustering heatmap showing the expression of hippocampal proteins in the control, 5**×**FAD, and MSP groups. **(E)** Volcano plots showing the significance and fold change of identified proteins between the 5**×**FAD vs. control and 5**×**FAD vs. MSP groups (FC > 1.1 or FC < 0.91, unpaired two-sided Student's *t*-tests, *p* < 0.05). **(F)** Venn diagram summarizing differential and overlapping proteins between the 5**×**FAD vs. control and 5**×**FAD vs. MSP groups (FC > 1.1 or FC < 0.91, unpaired two-sided student's *t*-tests, *p* < 0.05). **(G)** Clustering of differentially expressed proteins (DEPs) into two clusters using the Fuzzy C-mean (FCM) clustering algorithm. Proteomic analysis was performed on hippocampal tissues from 3 mice per group (*n* = 3). MSP denotes the low-dose MSP treatment group.

To explore the molecular mechanisms underlying the effects of MSP, we performed a quantitative proteomic analysis using Data-Independent Acquisition (DIA) technology on hippocampal tissues from control, 5 × FAD, and MSP-treated mice. Since no significant differences were observed between the low-dose and high-dose MSP groups, we selected the low-dose group for further analysis. In total, 7,476 proteins were identified, with 7,361 proteins providing quantitative data in at least 60% of the samples (Figure 5A). To minimize variability from differences in sample injection amounts, we applied median normalization to the proteomic data (Figure 5B). The distribution of protein abundance was consistent across all samples, indicating good parallelism. The median coefficient of variation (CV) for the three replicate samples in both the 5 × FAD and MSP groups was below 0.1 (Figure 5C), demonstrating excellent quantitative reproducibility. Clustering analysis of DEPs revealed significant differences between the 5 × FAD vs. control and 5 × FAD vs. MSP groups, with 500 and 253 DEPs identified, respectively (fold change >1.5 or < 0.67; *p* < 0.05 using Tukey's test; Figure 5D). A Venn diagram identified 41 DEPs consistently dysregulated in both 5 × FAD vs. control and 5 × FAD vs. MSP comparisons (Figures 5E–F). Fuzzy c-means (FCM) clustering analysis identified 115 DEPs that were upregulated (Cluster 1) and 31 DEPs that were downregulated (Cluster 2) in the 5 × FAD group. Importantly, these changes were largely reversed to baseline levels following MSP treatment (Figure 5G).

### 3.5 MSP inhibits ACSL4 expression in 5 × FAD mice

**Figure 6:**
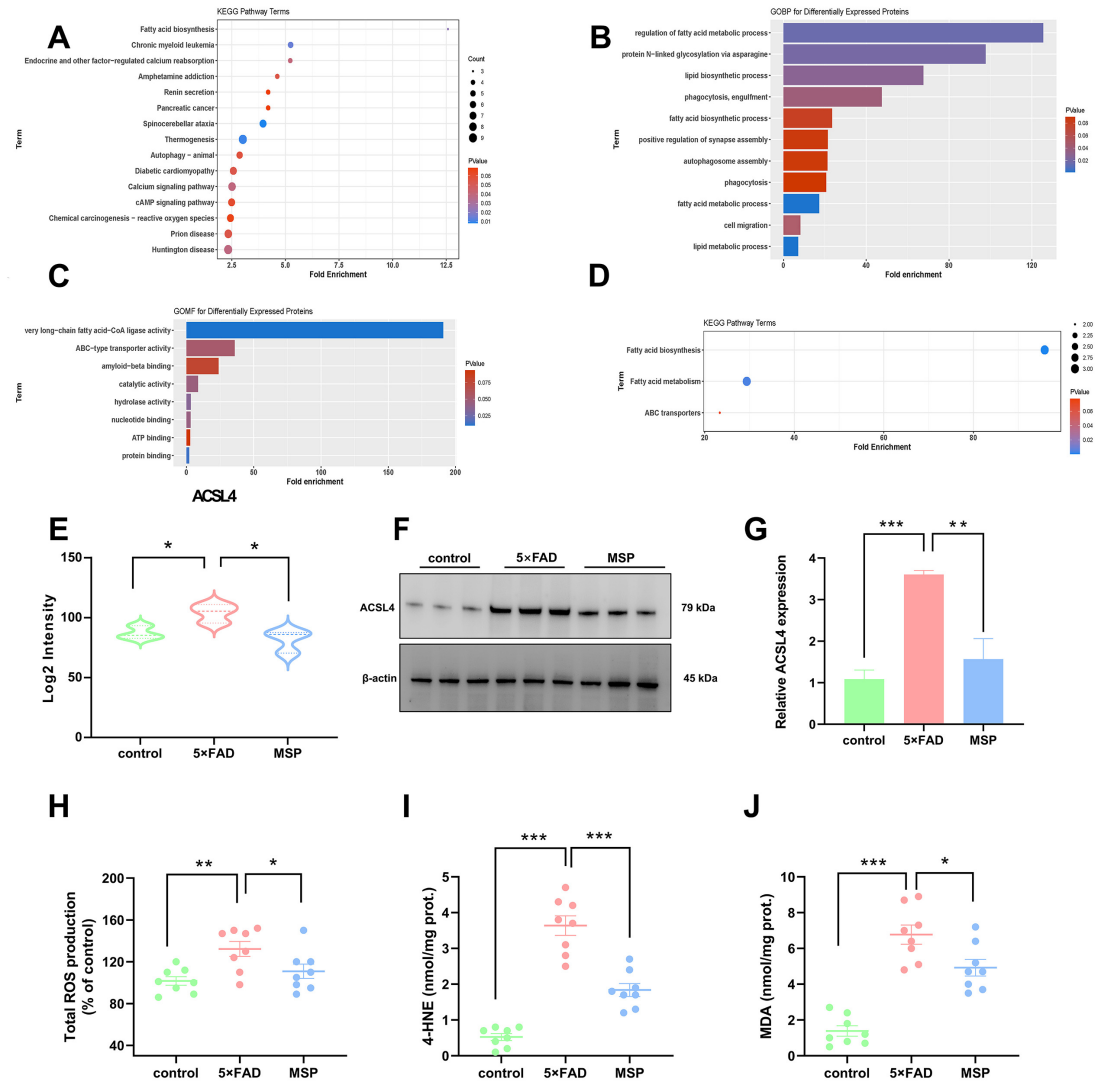
MSP treatment reduces ACSL4 expression and lipid peroxidation in 5 × FAD mice. **(A)** KEGG pathway enrichment analysis of DEPs from the 5**×**FAD vs. MSP comparison. **(B–D)** Enrichment analysis of Cluster 1 proteins using Gene Ontology (GO) biological process (GOBP), GO molecular function (GOMF), and KEGG pathways. **(E)** Box plots depicting relative protein expression levels of ACSL4 in each group. **(F, G)** Western blot analysis showing ACSL4 protein expression in hippocampal tissues from each group, with β-actin was used as a loading control. **(H)** Total reactive oxygen species (ROS) production in each group. **(I)** 4-hydroxynonenal (4-HNE) level in each group. **(J)** Malondialdehyde (MDA) levels in each group. Data are presented as mean ± SEM (*n* = 8 per group). *p < 0.05, **p < 0.01, ***p < 0.001.

Proteomic analysis revealed that MSP treatment modulates lipid metabolism and oxidative stress pathways in 5 × FAD mice, as evidenced by KEGG enrichment of DEPs associated with fatty acid biosynthesis and ROS regulation (Figure 6A). GO analysis further highlighted processes linked to lipid biosynthesis and very long-chain fatty acid-CoA ligase activity (Figures 6B–D). Among the DEPs, key proteins implicated in lipid homeostasis and redox balance were identified, including FASN (a central regulator of lipid synthesis), SLC25A24 (a mitochondrial transporter involved in energy and redox metabolism), ISCU (critical for iron-sulfur cluster assembly and antioxidant defense), and SPRYD7 (a potential modulator of synaptic plasticity and stress signaling). These findings suggest MSP coordinately targets lipid anabolism (FASN, ACSL4) and oxidative resilience (SLC25A24, ISCU), with ACSL4 emerging as a central hub protein bridging these pathways. Notably, ACSL4 expression was significantly elevated in 5 × FAD mice but robustly suppressed by MSP treatment (Figure 6E), underscoring its pivotal role in mediating MSP's lipid-regulatory effects. Western blot analysis of brain tissue confirmed these findings, showing that ACSL4 expression was upregulated in the 5 × FAD group and significantly decreased after MSP treatment (Figures 6F, G). These results suggest that MSP modulates ACSL4 expression, underscoring its pivotal role in mediating MSP's lipid-regulatory effects.

### 3.6 MSP suppresses lipid peroxidation in 5 × FAD mice

We also assessed the effect of MSP on lipid peroxidation. Consistent with our proteomic findings, MSP treatment significantly reduced ROS production in the brains of 5 × FAD mice (Figure 6H). Furthermore, MSP treatment effectively lowered the levels of lipid peroxidation products, such as MDA and 4-HNE, which are key biomarkers of lipid oxidative damage (Figures 6I–J). These results suggest that MSP mitigates lipid peroxidation in the 5 × FAD model, potentially providing neuroprotective effects by reducing oxidative stress. Overall, these results highlight potential therapeutic effects of MSP in modulating lipid metabolism and reducing oxidative damage in AD.

## 4 Discussion

Alzheimer's disease (AD) remains a pressing public health issue, with current treatments offering symptomatic relief without altering disease progression (1, 40). In this study, we evaluated the neuroprotective properties of MSP, a medicine-food homology formulation, in the 5 × FAD mouse model. We focused on its influence on lipid metabolism and oxidative stress—pathways highly relevant to nutritional interventions. Our integrated behavioral, histological, and proteomic approaches demonstrate that MSP alleviates cognitive decline, reduces Aβ and tau pathology, and modulates key molecular events in AD.

MSP significantly improved cognitive performance in AD mice, consistent with earlier reports of its neuroprotective benefits. Notably, it reduced Aβ plaque burden and tau hyperphosphorylation, indicating potential disease-modifying effects. MSP also suppressed microglial activation and lowered pro-inflammatory cytokines (IL-1β, TNF-α, and IL-6), supporting its role as a multi-target dietary supplement capable of mitigating neuroinflammation.

We also employed quantitative proteomic analysis to assess the molecular mechanisms underlying the effects of MSP. This analysis revealed that MSP treatment led to differential expression of 253 proteins in the hippocampus, the memory hub of the brain. Many of these proteins, especially those involved in fatty acid biosynthesis and ROS regulation, were significantly enriched. Among these DEPs, we prioritized ACSL4 for mechanistic validation based on three key considerations. First, ACSL4 serves as a metabolic checkpoint that bridges lipid metabolism and oxidative stress, two interconnected pathways prominently altered in our proteomic profile. Second, emerging evidence positions ACSL4 as a pivotal regulator of ferroptosis, a lipid peroxidation-driven cell death process implicated in neurodegenerative disorders (10, 41). Third, bioinformatic re-analysis of our proteomic dataset revealed that, unlike ACSL4, other ACSL family members (e.g., ACSL1, ACSL3, ACSL5, and ACSL6) did not show consistent or significant alterations, suggesting a distinct and prioritized role for ACSL4 in MSP's mechanism of action.

ACSL4, a protein involved in converting long-chain fatty acids into CoA derivatives essential for membrane synthesis and cellular homeostasis, was notably downregulated by MSP treatment. Dysregulation of ACSL4 has been linked to lipid imbalance, oxidative damage, and inflammation in neurodegenerative diseases (42). This context-dependent duality, where pathological overexpression drives neurodegeneration while physiological levels maintain membrane integrity, makes ACSL4 particularly notable as a potential mediator of MSP's neuroprotective effects. However, the correlative nature of our findings must be emphasized; the observed association between ACSL4 downregulation and phenotypic improvement, while compelling, does not establish causality. Future studies employing ACSL4-specific inhibitors (e.g., Rosiglitazone) or genetic knockdown/overexpression models *in vitro* and *in vivo* are essential to definitively validate its causal role.

Moreover, MSP treatment reduced the levels of byproducts of lipid peroxidation, such as MDA and 4-HNE. Considering that lipid peroxidation is a major contributor to neuronal dysfunction and death in AD (43), our results suggest that MSP's ability to reduce ROS production and lipid peroxidation products likely plays a central role in its therapeutic effects. This finding is particularly significant, as oxidative stress and lipid peroxidation exacerbate amyloidogenesis and tau hyperphosphorylation, leading to a vicious cycle of neurodegeneration (44, 45).

The formulation of MSP is complexity, inherent in its six-component composition, poses significant challenges for standardization and mechanistic dissection. Future research strategies should consequently extend beyond the current metabolite profiling to incorporate spectrum-effect relationship analysis and network pharmacology for identifying principal active constituents and putative synergies. It is also relevant to note that the applied dose, while substantial from a standard pharmacological viewpoint, is consistent with ranges encountered for crude herb-based dietary supplements, aligning with its medicine-food homology designation. Concerning tolerability, the absence of overt adverse effects during the 30-day intervention suggests preliminary safety, yet formal toxicological evaluation is a prerequisite for clinical advancement. Finally, the definitive development pathway for MSP—as a botanical drug or a nutraceutical—presents distinct regulatory frameworks that must be strategically addressed.

Nevertheless, several limitations must be acknowledged. The 30-day treatment duration in 7-month-old mice, while sufficient to demonstrate a conceptual therapeutic potential, may not fully capture long-term, disease-modifying effects. The choice of this age was based on established literature confirming robust Aβ pathology and cognitive deficits in 5 × FAD mice by 7 months (46, 47), making it a relevant timepoint for intervention. Future studies should include longer treatment periods and, crucially, post-treatment withdrawal phases to assess the persistence of benefits. The 5 × FAD model does not fully recapitulate human AD, particularly in late stages.

For future research, priority should be given to identifying active metabolites within MSP, establishing quality control standards, and validating ACSL4's role through genetic or pharmacological approaches. Clinical trials should assess MSP's safety, bioavailability, and efficacy as a dietary supplement in early AD populations, particularly through nutrition-oriented outcome measures such as lipid profiles, oxidative stress markers, and inflammatory cytokines. MSP exemplifies the potential of medicine-food homology interventions in modulating AD through nutrient-sensitive pathways. Its multifaceted effects encourage further development as a complementary nutritional strategy aimed at delaying progression and improving quality of life in AD.

## 5 Conclusion

This study demonstrates that MSP, a medicine-food homology formula, confers neuroprotection in a 5 × FAD Alzheimer's model. MSP improved cognitive function, reduced Aβ and tau pathology, and attenuated neuroinflammation. Proteomic analysis revealed that MSP modulates lipid metabolism and oxidative stress pathways, notably by downregulating ACSL4, a key regulator of lipid peroxidation. Consistent with this, MSP significantly decreased levels of oxidative damage markers. These findings support MSP as a promising dietary intervention for AD. Further clinical studies should evaluate its long-term efficacy and potential as complementary nutritional therapy. Future research may also explore synergistic effects with conventional treatments and validate relevant nutritional biomarkers.

## Data Availability

The mass spectrometry proteomics data generated in this study have been deposited to the ProteomeXchange Consortium via the iProX partner repository with the dataset identifier PXD072435: https://proteomecentral.proteomexchange.org/cgi/GetDataset?ID=PXD072435 (48, 49).

## References

[1] Scheltens P De Strooper B Kivipelto M Holstege H Chetelat G Teunissen CE . Alzheimer's disease. Lancet. (2021) 397:1577–90. doi: 10.1016/S0140-6736(20)32205-4 33667416 PMC8354300

[2] Andrade-Guerrero J Santiago-Balmaseda A Jeronimo-Aguilar P Vargas-Rodriguez I Cadena-Suarez AR Sanchez-Garibay C . Alzheimer's disease: an updated overview of its genetics. Int J Mol Sci. (2023) 24:3754. doi: 10.3390/ijms24043754 36835161 PMC9966419

[3] Rawat P Sehar U Bisht J Selman A Culberson J Reddy PH. Phosphorylated tau in Alzheimer's disease and other tauopathies. Int J Mol Sci. (2022). 23:12841. doi: 10.3390/ijms232112841 36361631 PMC9654278

[4] Liu E Zhang Y Wang JZ. Updates in Alzheimer's disease: from basic research to diagnosis and therapies. Transl Neurodegener. (2024) 13:45. doi: 10.1186/s40035-024-00432-x 39232848 PMC11373277

[5] Yin F. Lipid metabolism and Alzheimer's disease: clinical evidence, mechanistic link and therapeutic promise. FEBS J. (2023) 290:1420–53. doi: 10.1111/febs.16344 34997690 PMC9259766

[6] Zha X Liu X Wei M Huang H Cao J Liu S . Microbiota-derived lysophosphatidylcholine alleviates Alzheimer's disease pathology via suppressing ferroptosis. Cell Metab. (2024) 37:169–86.e9. doi: 10.1016/j.cmet.2024.10.006 39510074

[7] Mares J Costa AP Dartora WJ Wartchow KM Lazarian A Bennett DA . McIntire, brain and serum lipidomic profiles implicate lands cycle acyl chain remodeling association with APOEepsilon4 and mild cognitive impairment. Front Aging Neurosci. (2024) 16:1419253. doi: 10.3389/fnagi.2024.1419253 38938596 PMC11210445

[8] Fernandez RF Ellis JM. Acyl-CoA synthetases as regulators of brain phospholipid acyl-chain diversity. Prostaglandins Leukot Essent Fatty Acids. (2020) 161:102175. doi: 10.1016/j.plefa.2020.102175 33031993 PMC8693597

[9] Dattilo MA Benzo Y Herrera LM Prada JG Lopez PF Caruso CM . Regulation and role of Acyl-CoA synthetase 4 in glial cells. J Steroid Biochem Mol Biol. (2021) 208:105792. doi: 10.1016/j.jsbmb.2020.105792 33246155

[10] Zha X Liu X Wei M Huang H Cao J Liu S . Microbiota-derived lysophosphatidylcholine alleviates Alzheimer's disease pathology via suppressing ferroptosis. Cell Metab. (2025) 37:169–86 e9. 39510074 10.1016/j.cmet.2024.10.006

[11] Haney MS Palovics R Munson CN Long C Johansson PK Yip O . APOE4/4 is linked to damaging lipid droplets in Alzheimer's disease microglia. Nature. (2024) 628:154–61. doi: 10.1038/s41586-024-07185-7 38480892 PMC10990924

[12] Huang M Cheng S Li Z Chen J Wang C Li J . Preconditioning exercise inhibits neuron ferroptosis and ameliorates brain ischemia damage by skeletal muscle-derived exosomes via regulating miR-484/ACSL4 Axis. Antioxid Redox Signal. (2024) 41:769–92. doi: 10.1089/ars.2023.0492 38545792

[13] Zhuo B Qin C Deng S Jiang H Si S Tao F . The role of ACSL4 in stroke: mechanisms and potential therapeutic target. Mol Cell Biochem. (2024) 480:2223–46. doi: 10.1007/s11010-024-05150-6 39496916 PMC11961533

[14] Sultana R Butterfield DA. Oxidative modification of brain proteins in Alzheimer's disease: perspective on future studies based on results of redox proteomics studies. J Alzheimers Dis. (2013) 33(Suppl 1):S243–51. doi: 10.3233/JAD-2012-129018 22683528

[15] Siegel SJ Bieschke J Powers ET Kelly JW. The oxidative stress metabolite 4-hydroxynonenal promotes Alzheimer protofibril formation. Biochemistry. (2007) 46:1503–10. doi: 10.1021/bi061853s 17279615 PMC2530822

[16] McManus RM Latz E. NLRP3 inflammasome signalling in Alzheimer's disease. Neuropharmacology. (2024) 252:109941. doi: 10.1016/j.neuropharm.2024.109941 38565393

[17] Sun E Motolani A Campos L Lu T. The pivotal role of NF-kB in the pathogenesis and therapeutics of Alzheimer's disease. Int J Mol Sci. (2022) 23:8972. doi: 10.3390/ijms23168972 36012242 PMC9408758

[18] Maheshwari S. Ferroptosis signaling pathways: Alzheimer's disease. Horm Metab Res. (2023) 55:819–26. doi: 10.1055/a-2084-3561 37257500

[19] Deng C Chen H Meng Z Meng S. Roles of traditional chinese medicine regulating neuroendocrinology on AD treatment. Front Endocrinol. (2022) 13:955618. doi: 10.3389/fendo.2022.955618 36213283 PMC9533021

[20] Jieru H Lei F Tao L. Review on modified san jia san for the treatment of Alzheimer's disease. China J Chin Med. (2013) 28:589–90.

[21] Lijian F Lili Y Haiting G Kangli X Tao L. The effect of modified San Jia San on the memory ability and Aβ, NGF expression in the hippocampus of Alzheimer's disease rats. Shandong Med J. (2017) 57:37–9.

[22] Xuefeng L Geng Z Tao L. The regulation of Gai Liang San Jia San in the signaling pathway of AD cell model on rats. Lishizhen Med Mater Med Res. (2015) 26:2305–7.

[23] Jinjuan W Geng Z Miao J Tao L. Effect of cerebrospinal fluid with drug-containing modified San-Jia-San decoction on IL-1α, IL-1β and IL-6 of hippocampal neurons model injury induced by Aβ25-35. World Sci Technol. (2014) 16:1005–9.

[24] Scott NE. Expanding our understanding of the role of microbial glycoproteomes through high-throughput mass spectrometry approaches. Glycoconj J. (2019) 36:259–66. doi: 10.1007/s10719-019-09875-1 31270739

[25] Jain AP Sathe G. Proteomics landscape of Alzheimer's disease. Proteomes. (2021) 9:13. doi: 10.3390/proteomes9010013 33801961 PMC8005944

[26] Tang XM Guo JL Chen L Ho PC. Application for proteomics analysis technology in studying animal-derived traditional Chinese medicine: a review. J Pharm Biomed Anal. (2020) 191:113609. doi: 10.1016/j.jpba.2020.113609 32966940

[27] Wan Y Sun W Yang J Wang H Wang W Ye W . The protective effect of traditional Chinese medicine Jinteng Qingbi granules on rats with rheumatoid arthritis. Front Pharmacol. (2024) 15:1327647. doi: 10.3389/fphar.2024.1327647 38545550 PMC10965689

[28] Webster SJ Bachstetter AD Nelson PT Schmitt FA Van Eldik LJ. Using mice to model Alzheimer's dementia: an overview of the clinical disease and the preclinical behavioral changes in 10 mouse models. Front Genet. (2014) 5:88. doi: 10.3389/fgene.2014.00088 24795750 PMC4005958

[29] Amaeze O Marques ES Wei W Lazzaro S Johnson N Varma MVS . Evaluation of Nigerian medicinal plants extract on human P-glycoprotein and cytochrome P450 enzyme induction: implications for herb-drug interaction. Curr Drug Metab. (2021) 22:1103–13. doi: 10.2174/1389200223666211216142904 34915831 PMC11694737

[30] Amaeze O Eng H Horlbogen L Varma MVS Slitt A. Cytochrome P450 enzyme inhibition and herb-drug interaction potential of medicinal plant extracts used for management of diabetes in Nigeria. Eur J Drug Metab Pharmacokinet. (2021) 46:437–50. doi: 10.1007/s13318-021-00685-1 33844145 PMC11774566

[31] Lee D Lee WS Lim S Kim YK Jung HY Das S . A guanidine-appended scyllo-inositol derivative AAD-66 enhances brain delivery and ameliorates Alzheimer's phenotypes. Sci Rep. (2017) 7:14125. doi: 10.1038/s41598-017-14559-7 29074878 PMC5658413

[32] Lee J Lee S Ryu YJ Lee D Kim S Seo JY . Vaccinia-related kinase 2 plays a critical role in microglia-mediated synapse elimination during neurodevelopment. Glia. (2019) 67:1667–79. doi: 10.1002/glia.23638 31050055

[33] Lu J Zhang C Lv J Zhu X Jiang X Lu W . Antiallergic drug desloratadine as a selective antagonist of 5HT(2A) receptor ameliorates pathology of Alzheimer's disease model mice by improving microglial dysfunction. Aging Cell. (2021) 20:e13286. doi: 10.1111/acel.13286 33369003 PMC7811850

[34] He LL Wang YC Ai YT Wang L Gu SM Wang P . Qiangji decoction alleviates neurodegenerative changes and hippocampal neuron apoptosis induced by D-galactose via regulating AMPK/SIRT1/NF-kappaB signaling pathway. Front Pharmacol. (2021) 12:735812. doi: 10.3389/fphar.2021.735812 34630111 PMC8495211

[35] Petralla S Saveleva L Kanninen KM Oster JS Panayotova M Fricker G . Increased expression of transferrin receptor 1 in the brain cortex of 5 × FAD mouse model of Alzheimer's disease is associated with activation of HIF-1 signaling pathway. Mol Neurobiol. (2024) 61:6383–94. doi: 10.1007/s12035-024-03990-3 38296900 PMC11339108

[36] Lv J Shen X Shen X Zhao S Xu R Yan Q . NPLC0393 from *Gynostemma pentaphyllum* ameliorates Alzheimer's disease-like pathology in mice by targeting protein phosphatase magnesium-dependent 1A phosphatase. Phytother Res. (2023) 37:4771–90. doi: 10.1002/ptr.7945 37434441

[37] Jia J Ning Y Chen M Wang S Yang H Li F . Biomarker changes during 20 years preceding Alzheimer's disease. N Engl J Med. (2024) 390:712–22. doi: 10.1056/NEJMoa2310168 38381674

[38] Qiao O Ji H Zhang Y Zhang X Zhang X Liu N. et al. New insights in drug development for Alzheimer's disease based on microglia function. Biomed Pharmacother. (2021) 140:111703. doi: 10.1016/j.biopha.2021.111703 34083109

[39] Tang JJ Huang LF Deng JL Wang YM Guo C Peng XN . Cognitive enhancement and neuroprotective effects of OABL, a sesquiterpene lactone in 5 × FAD Alzheimer's disease mice model. Redox Biol. (2022) 50:102229. doi: 10.1016/j.redox.2022.102229 35026701 PMC8760418

[40] Twarowski B Herbet M. Inflammatory processes in Alzheimer's disease-pathomechanism, diagnosis and treatment: a review. Int J Mol Sci. (2023) 24:6518. doi: 10.3390/ijms24076518 37047492 PMC10095343

[41] Zhu ZY Liu YD Gong Y Jin W Topchiy E Turdi S . Mitochondrial aldehyde dehydrogenase (ALDH2) rescues cardiac contractile dysfunction in an APP/PS1 murine model of Alzheimer's disease via inhibition of ACSL4-dependent ferroptosis. Acta Pharmacol Sin. (2022) 43:39–49. doi: 10.1038/s41401-021-00635-2 33767380 PMC8724276

[42] Chen F Kang R Liu J Tang D. The ACSL4 network regulates cell death and autophagy in diseases. Biology. (2023) 12:864. doi: 10.3390/biology12060864 37372148 PMC10295397

[43] Kao YC Ho PC Tu YK Jou IM Tsai KJ. Lipids and Alzheimer's disease. Int J Mol Sci. (2020) 21:1505. doi: 10.3390/ijms21041505 32098382 PMC7073164

[44] Somin S Kulasiri D Samarasinghe S. Alleviating the unwanted effects of oxidative stress on Abeta clearance: a review of related concepts and strategies for the development of computational modelling. Transl Neurodegener. (2023) 12:11. doi: 10.1186/s40035-023-00344-2 36907887 PMC10009979

[45] Balendra V Singh SK. Therapeutic potential of astaxanthin and superoxide dismutase in Alzheimer's disease. Open Biol. (2021) 11:210013. doi: 10.1098/rsob.210013 34186009 PMC8241491

[46] Raju RP Cai L Tyagi A Pugazhenthi S. Interactions of cellular energetic gene clusters in the Alzheimer's mouse brain. Mol Neurobiol. (2024) 61:476–86. doi: 10.1007/s12035-023-03551-0 37632678 PMC10843700

[47] Daini E Secco V Liao W Zoli M Vilella A. A regional and cellular analysis of the early intracellular and extracellular accumulation of Abeta in the brain of 5 × FAD mice. Neurosci Lett. (2021) 754:135869. doi: 10.1016/j.neulet.2021.135869 33857550

[48] Ma J Chen T Wu S Yang C Bai M Shu K . iProX: an integrated proteome resource. Nucleic Acids Res. (2019) 47:D12117. doi: 10.1093/nar/gky869 30252093 PMC6323926

[49] Chen T Ma J Liu Y Chen Z Xiao N Lu Y . iProX in 2021: connecting proteomics data sharing with big data. Nucleic Acids Res. (2021) 50:D15227. doi: 10.1093/nar/gkab1081 34871441 PMC8728291

