# Supplementary material for: Modified Sanjia Powder ameliorates cognitive impairment and exerts neuroprotective effects in 5 × FAD mice: insights from quantitative proteomics
Source: Front Nutr. 2026 Jan 16;12:1699142. doi: 10.3389/fnut.2025.1699142 (PMC12855056; doi:10.3389/fnut.2025.1699142)
Supplement: Supplementary file 1 [file Table_1.docx]

**Supporting Information for**

**Modified Sanjia Powder Ameliorates Cognitive Impairment and Exerts Neuroprotective Effects in 5×FAD Mice: Insights from Quantitative Proteomics**

**1. Supporting tables**

**2. Supporting figures**

**1. Supporting table**

Supplementary table 1. List of the selected MRM parameters, fragmentor and collision energy for each analyte measured by LC-MS/MS assay.

| Compound | t_R_/min | Q1 | Q3 | Collision energy/eV | S-Lens |
| --- | --- | --- | --- | --- | --- |
| Lysine | 0.77 | 147.0 | 84.1 | 14 | 40 |
| Histidine | 0.77 | 156.0 | 110.1 | 11 | 39 |
| Valine | 0.87 | 118.0 | 72.1 | 6 | 28 |
| Guanosine | 0.87 | 284.1 | 152.1 | 15 | 50 |
| Hypoxanthine | 0.87 | 137 | 137 | - | 40 |
| Emodin | 8.70 | 269 | 171 | 30 | 131 |
| Galangin | 9.58 | 269.1 | 225.1 | 30 | 156 |

Supplementary table 2. Linearity and contents of LC-MS/MS assay for Compound.

| Compound | Calibration curve | R^2^ | Content (μg/g MSP) |
| --- | --- | --- | --- |
| Lysine | y = 762451x + 556441 | 0.9961 | 240 |
| Histidine | y = 1E+06x + 952355 | 0.9924 | 164 |
| Valine | y = 474178x + 75661 | 0.9971 | 243 |
| Guanosine | y = 1E+06x + 246357 | 0.9913 | 140 |
| Hypoxanthine | y = 374356x + 82699 | 0.994 | 82 |
| Emodin | y = 455.34x + 863.93 | 0.9954 | 6693 |
| Galangin | y = 27452x + 45103 | 0.9996 | 378 |

**2. Supporting figures**


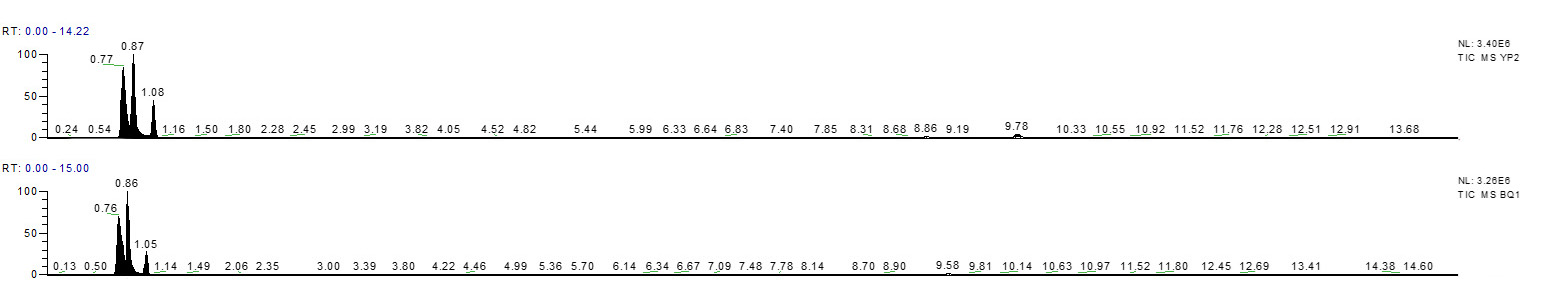


Supplementary figure 1: Total ion chromatogram of MSP and standard





Supplementary figure 2: Box plots depicting relative protein expression levels of ACSL1, ACSL3, ACSL5 and ACSL6 in each group.


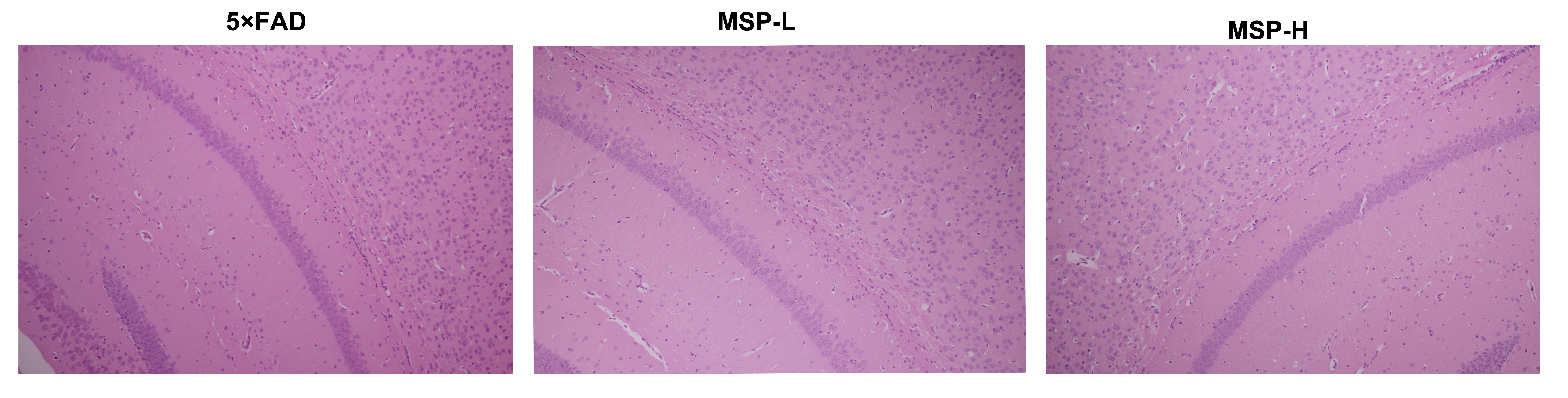


Supplementary figure 3: MSP treatment does not alter the overall brain structure in 5×FAD mice.
